# From Classroom to Career: The Impact of Guest Speakers on MSW Students’ Interest in Aging

**DOI:** 10.1093/geroni/igaf122.3266

**Published:** 2025-12-31

**Authors:** Mbita Mbao

**Affiliations:** Salem State University, Salem, Massachusetts, United States

## Abstract

The growing demand for geriatric social workers highlights the urgent need to attract more students to aging-related careers. However, many MSW students remain reluctant to enter this field. This study examined whether incorporating guest speakers from the aging services sector into social work curriculum could positively influence students’ attitudes and career intentions. Using a cross-sectional pre-posttest design, we surveyed 17 MSW students enrolled in a Social Work with Older Adults course. The Willingness to Work with Older People Scale was used to assess changes in students’ perceptions. Findings revealed statistically significant improvements in students’ confidence, perceived competence, and willingness to work with older adults. A key result was the increase in students’ agreement with the statement, “My professors advise me to consider aging career” (t = −3.31, p < .01), rising from a pretest mean of 3.59 (SD = 1.54) to a posttest mean of 4.88 (SD = 1.22). Additionally, Perceived Behavioral Control and Intention to Work with Older Adults measures showed significant gains, reflecting students’ increased readiness to enter the aging workforce.These findings suggest that guest speakers serve as powerful role models, bridging the gap between classroom learning and real-world practice. By exposing students to the realities and rewards of geriatric social work, guest speakers may play a critical role in shaping future workforce development.

